# Accumulative prediction values of serum thyroid stimulating hormone and visceral adipose tissue for metabolic syndrome in postmenopausal women: A 10‐year follow‐up study of Chinese population

**DOI:** 10.1111/1753-0407.13472

**Published:** 2023-09-25

**Authors:** Qiu Yang, Hongyi Cao, Qi Zeng, Bing Fu

**Affiliations:** ^1^ Department of endocrinology, Geriatric Diseases Institute of Chengdu, Center for Medical Research and Translation Chengdu Fifth People's Hospital Chengdu China; ^2^ Information center Chengdu Fifth People's Hospital Chengdu China; ^3^ Department of Radiology, Geriatric Diseases Institute of Chengdu Chengdu Fifth People's Hospital Chengdu China

**Keywords:** metabolic syndrome, thyroid stimulating hormone, visceral fat area, magnetic resonance imaging, postmenopausal

## Abstract

**Aims:**

We aim to explore the cumulative predictive value of elevated serum thyroid stimulating hormone (TSH) and visceral fat area (VFA) for metabolic syndrome (MS) development in postmenopausal women.

**Methods:**

A total of 1006 postmenopausal females were enrolled in a 10‐year prospective longitudinal study from 2011 to 2021 in the community of Banknote Printing Company of Chengdu. The sociodemographic information collection and anthropometric measurements were made by a professional nurse. Fasting blood samples were drawn for chemical analysis of fasting plasma glucose, triglycerides, low‐density lipoprotein cholesterol, high‐density lipoprotein cholesterol, and TSH.

Magnetic resonance imaging was performed to measure VFA. All the participants were categorized into four groups according to median VFA and serum level of TSH.

**Results:**

A total of 793 postmenopausal females without MS underwent a 10‐year follow‐up study grouping by TSH and VFA: Group 1 (TSH level <4.2 μIU/mL, and VFA < 70 cm^2^), Group 2 (TSH level ≥4.2 μIU/mL, and VFA < 70 cm^2^), Group 3 (TSH level <4.2 μIU/mL, and VFA ≥70 cm^2^) and Group 4 (TSH level ≥4.2 μIU/mL, and VFA ≥70 cm^2^). During the 10‐year follow‐up, MS was newly developed in 326 (41.1%) subjects. The incidence of MS was 29.8% (*n* = 53), 35.2% (*n* = 63), 41% (*n* = 87), and 55% (*n* = 123) from Group 1 to Group 4 (Group 4 vs other groups, *p* < .001). Cox regression analysis for MS prediction demonstrated that both TSH (Model 3, hazard ratio [HR] = 1.07 [95% confidence interval, 1.05–1.09]) and VFA (Model 4, HR = 1.02 [95% confidence interval, 1.01–1.08]) were not only independent predictors of MS but also involved some interaction between each other (*p* for interaction = .021).

**Conclusion:**

Our findings suggested that mutual interaction between higher TSH and VFA contributed to the development of MS. Further studies are needed to clarify these contributions.

## Graphical abstract

Highlights

- Elevated thyroid stimulating hormone (TSH) and visceral fat area (VFA) were critical factors for the prevalence of MS during postmenopause independently
- The concomitant elevated VFA and serum TSH levels were indeed a menopause‐related phenomenon that interacted with each other and appeared to co‐contribute to the increased risk of MS during the postmenopausal period

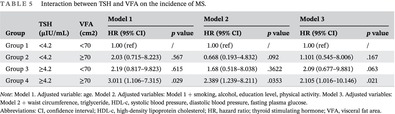

## 1 INTRODUCTION

Metabolic syndrome (MS) is a cluster of insulin resistance, central obesity, dyslipidemia, and hypertension that has become a major global health challenge along with increasing aging and urbanization.^1^

The worldwide prevalence of MS was reported to be 20%–25% according to different diagnostic criteria, various clinical conditions of female respondents, socioeconomic and environmental differences, genetic factors, lifestyle, and ethnic differences.^2^, ^3^, ^4^, ^5^, ^6^ More severely, the incidence of MS increased significantly in menopause and ranges from 31% to 69% globally.^7^, ^8^ Menopause is a critical period of complex sex steroid hormone fluctuations in females, resulting in a significant number of physiological changes^9^ with complete (or near‐complete) metabolic slowdown and a raised prevalence of MS.^10^ Thus these data indicated the urgent need for special attention to and prevention of MS after menopause.

It was reported that MS might be driven by the pathophysiological changes of estrogen deficiency, follicle‐stimulating hormone (FSH) elevation, and relative increase of testosterone in menopause.^11^ Aside from these well‐known factors, thyroid dysfunction^12^, ^13^, ^14^ and redistribution of body fat^15^ along with gonadal failure were well‐known drivers of MS. First, menopause increased total body fat and the redistribution of visceral adiposity.^16^, ^17^, ^18^ Second, the serum concentrations of thyroxine‐binding globulin and FSH increased in the menopausal period,^19^ resulting in an elevation of serum level of thyroid stimulating hormone (TSH) within or above the higher limits.

Accordingly, it can be inferred that sex hormone‐dependent visceral adiposity and TSH were the link between menopause and MS. Moreover, extant evidence supports that TSH and abdominal obesity may interact differently.^20^, ^21^ TSH independently regulates the lipolysis/lipogenesis balance, and adipose tissue could affect the activity of the hypothalamic–pituitary–thyroid axis system through adipokines release. However, the accumulative prediction values of TSH and central obesity for developing MS in postmenopause remain to be elucidated. Good health is critical because women will spend more than one third of their lives postmenopausal as life expectancy increases. As such, understanding this complex interplay of obesity, thyroid function, and menopause is important to providing the right advice and management. The present study (a) ascertained the morbidity of MS and the change of metabolic status with different baseline levels of TSH and visceral fat area (VFA), and (b) evaluated whether the combination of the two factors would increase the risk of MS in postmenopausal women.

## 2 MATERIALS AND METHODS

### 2.1 Study population

A 10‐year prospective study was conducted in the employee community of Banknote Printing Company of Chengdu from 2011 to 2021. It was a large community with >10 000 residents. All employees underwent a medical examination every 2 years. Simultaneously, we recruited postmenopausal females aged 45–65 in the 2011 physical examination. A professional nurse was responsible for sociodemographic information collection covering medical histories, consumption of smoking and alcohol, educational status, and physical activity. Subsequently, anthropometric measurements were performed, including height, weight, and waist circumference (WC). Serum fasting plasma glucose (FPG), triglycerides (TG), high‐density lipoprotein cholesterol (HDL‐c), and TSH were obtained at baseline and 10 years after in 2021. The exclusion criteria for baseline were as follows: unnatural menopause, estrogen progesterone replacement therapy, malignant tumor, cardiovascular disease, mental disorders, and MS. Meanwhile, women with data missing, who exited the follow‐up, and used drugs for hypertension, diabetes, and hyperlipidemia were also excluded during the follow‐up. This study was approved by Chengdu Fifth Hospital's ethics committee (NO. 2011‐031〔science〕‐01) and followed the Declaration of Helsinki. All patients gave informed consent.

### 2.2 Clinical and biochemical measurements

All the subjects submitted to a physical examination including height, weight, and WC measurement with unlined clothes and bare feet. WC was assessed at the horizontal circumference between the midline of the iliac crest and the lower edge of the 12th rib. Body mass index (BMI) was calculated as weight [kg]/height [m^2^]. After the participant sat for 15 minutes, the blood pressure of the brachial artery in the right arm was measured with a mercury sphygmomanometer three times, with intervals of more than 5 min, and the mean value was obtained.

Blood samples were drawn at 8 ~ 10 a.m. after 12 hours of fasting. The serum FPG, TG, low‐density lipoprotein cholesterol, and HDL‐c were measured by an Olympus AU 5400 automatic biochemical analyzer (Japan). Serum TSH was measured by enzyme linked immunosorbent assay (Bayer Co., Tokyo, Japan) (reference range: 0.1–4.2 μIU/mL). The intra‐assay and total coefficients of variation for the TSH assay were 4.5%–11.5% and 5.9%–11.9%, respectively.

### 2.3 Magnetic resonance imaging quantification of the visceral fat area

By using a 1.5‐T scanner (Magnetom Open Viva, Siemens AG, Erlangen, Germany) magnetic resonance imaging (MRI) data were obtained. The quantification of abdominal fat was detected at the level of the L_3_–L_4_ discs. SliceOmatic image analysis software (version 4.3, Tomovision, Montreal, Canada) was used to analyze MRI slices. VFA (cm^2^) were measured separately by two observers. The coefficient of variation from duplicate measurements of observers was 3.21% (1.05%–6.77%). A third observer conducted a repeated measurement to assess the repeatability. All images were analyzed by blinded independent experienced readers, unaware of the clinical characteristics of study subjects.^22^

### 2.4 Definition of metabolic syndrome

The 2005 International Diabetes Federation (IDF) ethnicity‐specific definitions of MS for Asian people was used^23^: central obesity (Chinese women WC ≥80 cm) plus any two: (a) raised TG >1.7 mmol/L or specific treatment for this lipid abnormality; (b) reduced HDL‐c 1.29 mmol/L or specific treatment for this lipid abnormality; (c) raised systolic blood pressure (SBP) ≥130 mm Hg, diastolic blood pressure (DBP) ≥85 mm Hg, or treatment of previously diagnosed hypertension; and (d) raised FPG≥5.6 mmol/L or previously diagnosed type 2 diabetes.

### 2.5 Statistical analysis

The normality examination of each variable was performed by the Kolmogorov–Smirnov test. Normally distributed continuous variables were described by mean and SD, median and quartile (25th percentile, 75th percentile) were used to describe the nonnormally distributed data. First, the comparison between the MS group and non‐MS group at the final visit was analyzed by Student's *t* test. Mann–Whitney *U* test was used for nonnormally distributed data. Second, in stratified analysis, we investigated differences for median VFA (70 cm^2^) and serum level of TSH (4.2 μIU/mL). The one‐way analysis of variance (normal distributions) and Kruskal–Wallis test (nonnormal distributions) were used to compare the difference in the metabolic components among the four groups. Additionally, post hoc Bonferroni contrast analysis was performed. Third, we used Cox regression model in estimating hazard ratios (HR) with 95% confidence intervals (CI) for MS risk, adjusting for covariates including smoking, alcohol, education level, physical activity, age, WC, TG, HDL‐c, SBP, DBP, and FPG. Model 1 was adjusted for age. Model 2 was adjusted for Model 1 + WC, TG, HDL‐c, SBP, DBP, and FPG. Model 3 was adjusted for Model 3 + VFA. Model 4 was adjusted for Model 3+ TSH. Fourth, the Wald and the likelihood ratio chi‐squared tests were performed to determine the interaction effect between TSH and VFA. Model 1 was adjusted for age. Model 2 was adjusted for Model 1 + smoking, alcohol, education level, physical activity. Model 3 was adjusted for Model 2 + WC, TG, HDL‐c, SBP, DBP, and FPG. *p*<.05 was considered statistically significant for all analyses.

## 3 RESULTS

### 3.1 Participants

**FIGURE 1:**
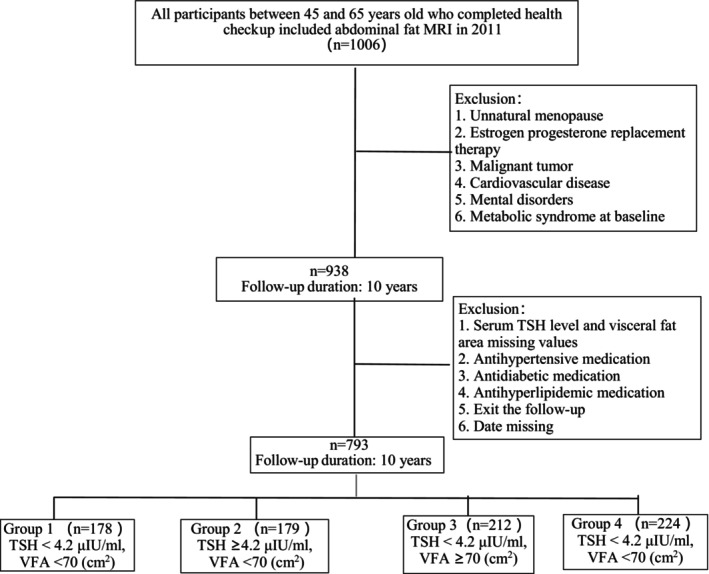
Study population. TSH, thyroid stimulating hormone; VFA, visceral fat area.

The flow chart shows the participants' selection in Figure 1. A total of 1006 participants were enrolled this study, and 68 were excluded according the exclusion criteria at baseline. The normal reference range of serum TSH in our laboratory was 0.1–4.2 μIU/mL. Thus serum level of TSH ≥4.2 μIU/mL would be defined as elevated and as one of the bases for grouping. Considering that there was no diagnostic criteria for normal VFA in postmenopausal women, we used the median of VFA of the present population as the other basis for grouping.

A total of 938 participants were categorized into four groups according to median VFA (70 cm^2^) and serum level of TSH (4.2 μIU/mL). The four groups were as follows: Group 1 was TSH level <4.2 μIU/mL and VFA < 70 cm^2^. Group 2 was TSH level ≥4.2 μIU/mL and VFA < 70 cm^2^. Group 3 was TSH level <4.2 μIU/mL and VFA ≥70 cm^2^. Group 4 was TSH level ≥4.2 μIU/mL and VFA ≥70 cm^2^. The final follow‐up loss rate was 15.46% (*n* = 145), and then 793 subjects finished the 10‐year follow‐up (Group 1, *n* = 178; Group 2, *n* = 179; Group 3, *n* = 212; Group 4, *n* = 224;). Table 1 showed the baseline characteristics of the participants. The mean age was 56.3 ± 7.4 years, ranging from 45 to 65 years. The mean BMI was 24.8 ± 2.2 kg/m^2^ and mean VFA was 70 ± 36.5 cm^2^. Compared with Groups 1 and 2, BMI and WC increased significantly in Groups 3 and 4 (*p* < .001). Also, the serum level of TG increased in Group 3 and Group 4 (*p* = .022), and FPG increased in Group 4 (*p* = 0.012). However, HDL‐c levels, SBP, and DBP showed no difference among the four groups.

**TABLE 1:** Baseline characteristics of participants.

Variable	Group 1 (*n* = 178)	Group 2 (*n* = 179)	Group 3 (*n* = 212)	Group 4 (*n* = 224)	*p* value
Age (years)	56 [52, 60]	55 [51, 59]	58 [54, 61]	57 [54, 62]	.101
TSH (μIU/mL)	3.9 ± 1.03^a^	4.9 ± 2.14^b^	4.09 ± 1.45^a^	4.89 ± 1.34^b^	<.001
VFA (cm^2^)	65.21 ± 21.83^a^	67.69 ± 23.15^a^	74.11 ± 25.07^b^	77.42 ± 24.15^b^	<.001
WC (cm)	77.11 ± 8.00^a^	79.87 ± 5.94^a^	88.8 ± 7.91^b^	88.35 ± 6.79^b^	<.001
BMI (kg/m^2^)	21.85 ± 2.67^a^	23.11 ± 2.13^a^	24.95 ± 2.61^b^	25.37 ± 2.63^b^	<.001
SBP (mm Hg)	118.65 ± 13.34	117.95 ± 13.21	118.16 ± 12.34	119.86 ± 12.72	.768
DBP (mm Hg)	73.71 ± 9.17	74.29 ± 9.15	73.17 ± 9.46	75.03 ± 9.5	.594
HDL‐c (mmol/L)	1.28 ± 0.38	1.33 ± 0.49	1.20 ± 0.52	1.18 ± 0.62	.056
TG (mmol/L)	2.09 [1.79, 2.88]^a^	1.99 [1.69, 2.72]^a^	2.45 [1.78, 3.04]^a,b^	2.78 [2.31, 3.11]^b^	.022
FPG (mmol/L)	5.14 ± 0.52^a^	5.67 ± 0.79^a^	5.91 ± 0.66^a^	6.45 ± 0.98^b^	.012

*Note*: Data are presented as the mean ± SD or (25 percentile, 75 percentile) for continuous variables. *p* < .05 means significant difference between a and b. Different superscripts (a and b) indicated differences between the two groups.

Abbreviations: BMI, body mass index; DBP, diastolic blood pressure; FPG, fasting plasma glucose; HDL‐c, high‐density lipoprotein; SBP, systolic blood pressure; TG, triglyceride; TSH, thyroid stimulating hormone; VFA, visceral fat area.

### 3.2 Incidence of metabolic syndrome and follow‐up

During the 10‐year follow‐up, MS was newly diagnosed in 326 (41.1%) patients. The incidence of MS was 29.8% (*n* = 53) in Group 1, 35.2% (*n* = 63) in Group 2, 41% (*n* = 87) in Group 3, and 55% (*n* = 123) in Group 4 (Group 4 vs other groups, *p <* .001). The comparisons of metabolic components between baseline and follow‐up in the study were shown in Table 2. Compared with the non‐MS group, WC and BMI were significantly higher in the MS group (*p <* .001). Additionally, the serum level of FPG was also higher (*p =* .031) and HDL‐c was lower (*p =* .013) in MS group.

**TABLE 2:** Comparison of characteristics of subjects between the baseline and 10‐year follow‐up.

Variable	Baseline values	After 10‐year follow‐up		*p* value
		MS	Non‐MS	
Number	793	326	467	/
WC (cm)	74 ± 11.06	89 ± 14.23	73 ± 13.82	<.001
BMI (kg/m^2^)	23.15 ± 6.59	27.15 ± 5.19	24.15 ± 6.59	<.001
SBP (mmHg)	120.7 ± 13.06	129.7 ± 14.16	122.2 ± 13.16	.494
DBP (mmHg)	80.77 ± 8.54	83.65 ± 10.41	81.15 ± 9.82	.161
HDL‐c (mg/dL)	1.38 ± 0.52	1.18 ± 0.34	1.22 ± 0.72	.031
TG (mg/dL)	1.89 [1.59, 2.15]	2.34 [1.89, 2.83]	1.99 [1.53, 2.21]	.263
FPG (mg/dL)	5.79 ± 0.87	7.18 ± 0.87	6.43 ± 0.76	.013

*Note*: Data are presented as the mean ± SD or (25 percentile, 75 percentile) for continuous variables. The data were analyzed by Student's *t* test for comparison of two groups.

Abbreviations: BMI, body mass index; DBP, diastolic blood pressure; FPG, fasting plasma glucose; HDL‐c, high‐density lipoprotein cholesterol; SBP, systolic blood pressure; TG, triglyceride; TSH, thyroid stimulating hormone; VFA, visceral fat area.

### 3.3 Comparison among characteristics by category of thyroid stimulating hormone and visceral fat area at baseline

Taking the groupings into consideration, the difference in metabolic components among the four groups was analyzed. The changes between baseline and the final study were also performed in Table 3. The follow‐up data and changes of WC and BMI showed significant differences among the groups (*p* < .05), where the highest levels were found in Group 4 and no significant differences were found among Group 1, Group 2, and Group 3. The level of FBG was higher in Group 3 and Group 4 than in Group 1 and Group 2 (*p* = .043); the change of FPG showed the same result (*p* = .014). Additionally, the alterations in TG levels were significantly different among the groups (*p* = .047), although the follow‐up data failed to show a significant difference (*p* = .265). Group 4 exhibited the largest increase in TG levels between the baseline and follow‐up. But it was not statistically significant for other factors of MS even though Group 4 had an increased trend of BP and a decreased trend of HDL‐c at the end of follow‐up.

**TABLE 3:** Follow‐up characteristics and their respective variations from the baseline.

Variable	Group 1 (*n* = 218)	Group 2 (*n* = 165)	Group 3 (*n* = 239)	Group 4 (*n* = 194)	*p* value
WC (cm)	78.11 ± 7.19^a^	81.87 ± 6.04^a^	89.91 ± 5.11^a,b^	91.15 ± 6.03^b^	<.0011
Δ WC (cm)	0.9 ± 0.18^a^	1.06 ± 0.27^a^	1.23 ± 0.41^a,b^	1.91 ± 0.38^b^	.010
BMI (kg/m^2^)	22.19 ± 2.45^a^	23.99 ± 3.01^a^	25.15 ± 2.93^a,b^	26.89 ± 2.32^b^	<.001
Δ BMI (kg/m^2^)	1.02 ± 0.61^a^	0.98 ± 0.46^a^	1.12 ± 0.31^a,b^	1.24 ± 0.44^b^	.020
SBP (mmHg)	121.7 ± 12.06	120.2 ± 11.56	119.7 ± 13.11	123.2 ± 14.38	.487
Δ SBP (mmHg)	1.77 ± 8.17	1.65 ± 9.71	1.58 ± 8.05	1.99 ± 10.07	.678
DBP (mmHg)	80.04 ± 7.41	80.99 ± 10.09	81.17 ± 9.94	82.76 ± 10.48	.562
Δ DBP (mmHg)	1.13 ± 5.17	1.92 ± 6.12	2.01 ± 5.97	3.18 ± 7.02	.711
HDL‐c (mmol/L)	1.44 ± 0.65	1.40 ± 0.51	1.28 ± 0.22	1.34 ± 0.66	.059
Δ HDL‐c (mmol/L)	0.37 [0.27, 0.96]	0.34 [0.19, 0.78]	0.41 [0.18, 0.88]	0.45 [0.14, 0.99]	.881
TG (mmol/L)	2.37 [1.89, 3.01]^a^	2.07 [1.61, 2.98]^a^	2.80 [1.99, 3.31]^a^	2.93 [2.02, 3.51]^b^	.265
Δ TG (mmol/L)	0.13 [−0.34, 0.52]^a^	0.22 [−0.14, 0.61]^a^	0.37 [−0.11, 0.91]^a^	0.41 [0.23, 1.01]^b^	.047
FPG (mmol/L)	5.51 ± 0.68^a^	5.98 ± 0.89^a^	6.06 ± 0.79^b^	7.11 ± 1.03^b^	.043
Δ FPG (mmol/L)	0.88 ± 0.25^a^	0.96 ± 0.47^a^	1.01 ± 0.79^b^	1.28 ± 0.93^b^	.014

*Note*: Data are presented as the mean ± SD or (25 percentile, 75 percentile) for continuous variables. *p* < .05 means significant difference between a and b. Different superscripts (a and b) indicated differences between the two groups.

Abbreviations: BMI, body mass index; DBP, diastolic blood pressure; HDL‐c, high‐density lipoprotein cholesterol; SBP, systolic blood pressure; TG, triglyceride. FPG, fasting plasma glucose; TSH, thyroid stimulating hormone; VFA, visceral fat area.

### 3.4 Cox regression analysis for metabolic syndrome

With MS as the dependent variable, Cox regression analysis was used to evaluate the independent variables of TSH and VFA (Table 4). The Cox regression analysis showed that baseline TSH and VFA were significant predictors after adjusting for age (Model 1) and all metabolic factors (WC, TG, HDL‐c, SBP, DBP, FPG) (Model 2). After adjusting for VFA, TSH remained an independent predictor for the new onset of MS (Model 3, HR = 1.07 [95% CI, 1.05–1.09]). In the TSH‐adjusted multivariate model, VFA was still an independent predictor of MS (Model 4, HR = 1.02 [95% CI, 1.01–1.08]). Compared to unadjusted VFA and TSH factors, Model 2 showed more significant HR than Model 3 (TSH, HR = 1.07 [95% CI, 1.05–1.09]) and Model 4 (VFA, HR = 1.02 [95% CI, 1.01–1.08]). Furthermore, these data suggested that TSH and VFA might associate with each other and co‐contribute to MS.

**TABLE 4:** Hazard ratios for incidence of metabolic syndrome according to baseline TSH and visceral obesity status.

Models	TSH ≥4.2 μIU/mL		VFA ≥70 cm^2^	
	HR (95% CI)	*p* ‐value	HR (95% CI)	*p* value
Model 1	1.04 (1.02–1.11)	<.001	1.04 (1.01–1.05)	.02
Model 2	1.09 (1.06–1.11)	<.001	1.12 (1.08–1.15)	<.001
Model 3	1.07 (1.05–1.09)	.005	/	/
Model4	/	/	1.02 (1.01–1.08)	.002

*Note*: Model 1. Adjusted variable: age. Model2. Adjusted variables: Model 1 + waist circumference, triglyceride, HDL‐c, systolic blood pressure, diastolic blood pressure, fasting plasma glucose. Model 3. Adjusted variable: Model 2+ VFA. Model 4. Adjusted variable: Model 2+ TSH.

Abbreviations: CI, confidence interval; HDL‐c, high‐density lipoprotein cholesterol; HR, hazard ratio; TSH, thyroid stimulating hormone; VFA, visceral fat area.

Table 5 showed the interactive analysis of the interactions between TSH and VFA on the incidence of MS in three models. The HR (95% CI) of MS showed a growth trend from group 1 to group 4 in model 1 and model 3. Compared with group 1, the HR in group 4 was 3.011 (1.106–7.315) in model 1, 2.389 (1.239–8.211) in model 2, and 2.105 (1.016–10.146) in model 3 respectively, which meant that group 4 had a 2–3 fold higher risk of MS. Additionally, model 3 revealed a significant interaction between TSH and VFA in relation to MS risk when adjusting age, sociodemographic factors, and components of MS (*p* for interaction = .021).

**TABLE 5:** Interaction between TSH and VFA on the incidence of MS.

Group	TSH (μIU/mL)	VFA (cm2)	Model 1		Model 2		Model 3	
			HR (95% CI)	*p* value	HR (95% CI)	*p* value	HR (95% CI)	*p* value
Group 1	<4.2	<70	1.00 (ref)	/	1.00 (ref)	/	1.00 (ref)	/
Group 2	<4.2	≥70	2.03 (0.715–8.223)	.567	0.668 (0.193–4.832)	.092	1.101 (0.545–8.006)	.167
Group 3	≥4.2	<70	2.19 (0.817–9.823)	.615	1.68 (0.518–8.038)	.3622	2.09 (0.677–9.881)	.063
Group 4	≥4.2	≥70	3.011 (1.106–7.315)	.029	2.389 (1.239–8.211)	.0353	2.105 (1.016–10.146)	.021

*Note*: Model 1. Adjusted variable: age. Model 2. Adjusted variables: Model 1 + smoking, alcohol, education level, physical activity. Model 3. Adjusted variables: Model 2 + waist circumference, triglyceride, HDL‐c, systolic blood pressure, diastolic blood pressure, fasting plasma glucose.

Abbreviations: CI, confidence interval; HDL‐c, high‐density lipoprotein cholesterol; HR, hazard ratio; thyroid stimulating hormone; VFA, visceral fat area.

## 4 DISCUSSION

The main finding of the present study was that elevated TSH and VFA were critical factors for the prevalence of MS during postmenopause independently. Additionally, the interaction of VFA and TSH might further increase the risk of MS.

To the best of our knowledge, this was the longest follow‐up study concerning the cumulative predictive value of TSH and VFA on MS in the postmenopausal period. Previous studies had shown that TSH and central obesity changing with menopause and aging played a crucial role in the body's metabolism separately. Thus the prevalence of MS was 41.1% in our population, which was close to previous studies with prevalence between 43.2 and 61.5% with IDF criteria in postmenopausal women.^7^ The result was consistent with previous studies showing that the prevalence of MS was significantly higher in postmenopausal women than in other adults.^12^, ^13^

The associations between elevated TSH and MS are well described in adults.^24^, ^25^, ^26^ A cross‐sectional study^27^ of 2205 euthyroid postmenopausal women revealed an increased prevalence of MS with elevated TSH quartile. As there is a female preponderance in thyroid disorders, and mild TSH elevations can be indicators of physiological change,^28^, ^29^, ^30^ it remains controversial whether menopause affects the associations. We previously conducted a cross‐sectional study with a sample of 1000 participants of postmenopausal women (40–65 years). No differences were found between thyroid hormones and the number of MS components and the prevalence of MS. However, the relationship became apparent in this 10‐year follow‐up study. Because direct crosstalk was found between androgen and thyroid hormone axes, it can be inferred that the influence of menopause magnifies the relationship between increased TSH and MS. Nevertheless, studies on the relationship between menopause and thyroid function are few and do not allow to clarify the potential mechanism.

Notably, accumulated evidence have showed that fat accumulation shifted from subcutaneous to visceral because of hormonal changes (high levels of androgens versus estrogen)^31^, ^32^ during the menopausal transition,^33^, ^34^ resulting in increased central obesity after menopause.^20^, ^21^ WC, an indicator of central obesity,^35^ could not differentiate between subcutaneous fat and visceral fat, of which the latter is a source of proinflammatory cytokines that contribute to insulin resistance and cardiovascular and diabetes‐related mortality.^36^ Thus, we used MRI, which was one of the gold‐standard imaging techniques, to assess the visceral adipose tissue and showed that VFA was an independent predictor of MS. Previous studies have indicated that visceral fat was positively associated with fasting insulin, insulin response to an oral glucose challenge,^37^, ^38^ and the development of further disturbances associated with MS.^39^, ^40^, ^41^ The underlying physiological mechanisms have been intensely studied including hyperlipolytic,^42^, ^43^ poor response to insulin,^42^, ^44^ and dysregulation of adipocytokines.^45^

Additionally, the present study also found significant interactions between TSH and VFA and lead to 2–3 fold higher risk of MS in combination. Both cross‐sectional and longitudinal studies were devoted to associations between thyroid function within the normal range and obesity have been found. Previous studies showed adipose tissue per se might influence the release and function of TSH. The level of TSH increased as compensatory to the hypothalamus‐pituitary‐thyroid axis because the TSH receptor was downregulated in VFA in a population with morbid obesity.^46^ Wang et al^47^ found TSH was positively associated with both general and abdominal obesity in a longitudinal cohort study of 481 Chinese adolescent school‐aged girls in east China. A retrospective clinical study of Chinese subjects with morbid obesity (BMI ≥32.5 kg/m^2^, *n* = 62) showed that TSH decreased notably after losing visceral adipose tissue by sleeve gastrectomy surgery.^48^ Our previous study yielded inconsistent results showing no association between TSH and abdominal obesity in a sample of euthyroid postmenopausal women.^49^ This discrepancy is likely due to a higher serum level of TSH and more serious metabolic disorders including central obesity during the follow‐up research, which would make the association obvious. The well‐known mechanisms are as follows. First, adipocytes not only derived leptin to stimulate the secretion of TSH^50^, ^51^ but also released abundant inflammatory cytokines to compromise the iodide uptake activity of thyroid cells.^52^, ^53^ Second, the expression of TSH receptors in adipose tissues, particularly visceral fat, is reduced and leads to the peripheral resistance of thyroid hormones. In contrast, elevated TSH could contribute to insulin resistance by inhibiting protein kinase B phosphorylation through binding to the TSH receptors expressed on the differentiated human adipocytes.^46^ Thus, the interaction between VFA and TSH might turn into a vicious circle for increasing the risk of MS.

As far as we know, this is the first population‐based study to ascertain the duplicate effects of body fat distribution measured by MRI techniques and serum TSH on the prevalence of MS in postmenopausal women. However, the present study also has some limitations. First, the conclusions should be consistently verified in multiple populations because it was a single‐center study with only Chinese postmenopausal women. Second, the loss to follow‐up bias should be taken into consideration, because 145 patients were unable to participate in the follow‐up assessment, which may have affected the study results. Third, although WC is insufficient to detect visceral fat exactly, it is widely used as a general indicator of abdominal obesity. We would use retrospective research in this prospective study to evaluate effective cutoffs of WC to predict MS by comparing WC with VFA.

## 5 CONCLUSION

In conclusion, the concomitant elevated VFA and serum TSH levels were indeed a menopause‐related phenomenon that interacts and appears to co‐contribute to the increased risk of MS during the menopausal status. More broadly, conferring the increased prevalence of MS in postmenopausal women, attention needs to be paid to the development of serum TSH and abdominal obesity in postmenopause period. Furthermore, the pathogenesis of the interaction between increased TSH and visceral adipose in postmenopausal women needs to be fully explored to unveil new treatments for MS in the future.

## AUTHOR CONTRIBUTIONS

All authors contributed equally to the literature search, study design, data collection, and data interpretation. Qiu Yang conceptualized and designed the study and drafted the manuscript. Hongyi Cao reviewed and revised the manuscript and approved the final manuscript as submitted. Qi Zeng conducted statistical analysis/interpretation. Bing Fu undertook data acquisition and supervised all aspects of the study.

## CONFLICT OF INTEREST STATEMENT

The authors have declared no conflicts of interest.
